# PD04 A Systematic Review Of Decision Analytical Modeling Studies Of Medicines In The Middle East

**DOI:** 10.1017/S0266462324002745

**Published:** 2025-01-07

**Authors:** Zainab Abdali, Tuba Saygin Avsar, Sue Jowett, Muslim Abbas Syed, Khalifa Elmusharaf, Louise Jackson

## Abstract

**Introduction:**

Economic evaluation using decision analytical models (DAMs) plays a limited role in shaping healthcare resource optimization and reimbursement decisions in the Middle East. This review aimed to systematically examine economic evaluation studies focusing on DAMs of medicines in the Middle East, defining methodological characteristics and appraising the quality of the identified models.

**Methods:**

Six databases were searched (MEDLINE, Embase, EconLit, Web of Science, the Global Health Cost-Effectiveness Analysis Registry, and the Global Index Medicus) from 1998 to September 2023 to identify published DAMs of medicines in the Middle East. Studies meeting the inclusion criteria—full economic evaluations of medicines using a model-based method in the Middle East—were included. Data were extracted and tabulated to include study characteristics and methodological specifications. The results were analyzed narratively. The Philips checklist was used to assess the quality of the studies.

**Results:**

Sixty-two DAM studies of medicines were identified from nine Middle Eastern countries, the majority of which (76%) were conducted in Iran, Turkey, and Saudi Arabia. The cost effectiveness of medications for non-communicable diseases was explored in 70 percent of the models. Cost-effectiveness thresholds based on gross domestic product were commonly used. International sources provided data on intervention effectiveness and health outcomes, while national sources were mainly used for the costs of resource use. Most models incorporated an assessment of parameter uncertainty, whereas other types of uncertainty were not explored. Studies from high-income countries were generally of higher quality than those from middle-income countries.

**Conclusions:**

The number of published DAMs was low considering the available medicines and disease burden. Key aspects of high quality DAMs regarding model structure, input sources, and uncertainty assessment were not consistently fulfilled. Recommendations for future studies and policies included strengthening existing health economic capacities, establishing country-specific health technology assessment systems, and initiating collaborations to generate national cost and outcome data.

